# Uncleanliness

**Published:** 1861-01

**Authors:** Geo. S. Fouke

**Affiliations:** Westminster, Md.


Uncleanliness.—*By Geo. S. Fouke.*—Notwithstanding it may be regarded a little in the light of a pathological *extravaganza*—that Parmelian saying, viz : that " when teeth are kept literally clean, no disease will ever be perceptible," yet it must be acknowledged that structures so " simple, yet effective," as the teeth, can " be made subject to disease by no slight causes, independent of those which result from accidental mechanical injury."

*Uncleanliness* is reckoned amongst the first of all external or local causes of caries. Hence, it is really a serious question our patients often ask us—" Doctor, *how* shall I keep my teeth clean ?" It is a question the profession should reply to with earnest particularity ; for, simple as it is—a " literally clean " tooth—it is not a thing entirely undivested of difficulty and some misapprehension, and the practical realization of clean teeth, in immaculate maxillaries, seems to be of less easy accomplishment than the task of keeping our masticators " fastidiously " clean, in the *imagination*, which may be unfortunately filled with erroneous or very fanciful " early impressions " upon the important subject of attention to dental cleanliness. The subject of *brushing teeth* has received some attention in our journals lately, and some timely practical hints given in the Dental Cosmos renders it unnecessary to say much either for professional or popular enlightenment, as to how people should clean their teeth. The matter is getting to be understood. However, a few thoughts upon this subject may not be out of place in the *Examiner.* Now, it is the consentient voice of every dental practitioner, that cleanliness is a most effective prophylactic against caries, and that its reverse, viz., a want of cleanliness, is the great barrier to permanency of success in operative denistry. Much skillful, solid work of the operative dentist, is being constanly undone by the insidious yet powerful effect, not of positive, *disgusting*

uncleanliness, but of a faulty or defective hygiene. It is a dental truth that should be inculcated on all hands, that scrupulous attention to cleansing the teeth is more important *after* operation are performed, than *before*. No extraneous matters should be allowed to remain on surfaces which have been recently *filed*, or where cavities have been *plugged*. Faultiness or defectiveness of dental cleanliness, may readily exist even where there is no little hard or vigorous rubbing of the teeth, and it is this want of *literal* cleanliness, that the observing practitioner recognizes as the undoer, very often, of his best operations and the powerful destroyer of many teeth upon which he expended much skill and labor to save from threatened *dissolution*.

To those persons who tell us "they brush their teeth from three to five times every day, using powders twice, and balm of a thousand flowers three times, and still they can not keep their teeth clean"—as well as to those who tell us "they brush hard and use the hardest brush they can get, and yet can not keep the *tartar off*," we would say, "stop that brushing" for awhile, and learn a little practical knowledge of the wants of your "ivory-tessellated halls." You need some *theory* and some *anatomical* ideas respecting the external structure of your teeth; respecting the position and arrangement of teeth in your jaws; respecting the dangerous and assailable points of the different classes of the dental organs. *Examine* your teeth once! Look at them in the places where nature has put them, and a brief study of their forms and localities will give you such a lesson of their simple, yet essential wants, that you will soon become intelligent and not "excessive tooth-brushers," effective and not "fastidious" tooth-cleansers. This lesson all must learn: first, that cleanliness is wanted on the crowns of the "jaw teeth," *i. e.*, on the grinding surfaces of the molars and bicuspids. The masticatory surfaces of these teeth, have fissures or indentations in them, more or less deep, where the enamel meets, as it were, in irregular folds. These are dangerous points. Here decay commences. These *sulci* must be kept clean. If the fissures are naturally deep, and there exists a carious chachexia or tendency to the development of caries, it will be hard to keep these places clean, and the best thing is to get some dentist to excavate these uncleansible cavities, and fill them up solidly with gold. These points once well filled up with gold, there will then be no use for *excessive* brushing on the grinding surfaces of the "jaw-teeth"—the friction of chewing will keep these parts of the teeth literally clean. The *second* point in the lesson to be learned, is this: that cleanliness is essential on all the lateral or side surfaces of the teeth. No extraneous matters, whatever, must be suffered to accumulate in the interstices between the front or the back teeth. These intermediary localities are assailable points, where decay is very liable to commence its ravages. The liability is greatest where the contact of the teeth is greatest, and where effective cleanliness is hindered, or rendered impracticable, perhaps, in consequence of irregular arrangement and over-crowdedness of the organs. It may be necessary in order to get the side surfaces of the entire denture in a condition to allow of keeping these points clean, to submit the denture to the care of the dental practitioner, who will institute hygienical treatment which may result in placing all the intermediary or lateral localities in a perfectly *cleansible* condition. The *third* item in the dental knowledge, essential to be known by all who would keep their teeth clean is this: the teeth all have "*necks*." The crowns of the teeth are all arched with beautiful festoons of rosy gum. Just around these festoons, and "a little under the margin of the gum" is the *cervex* or *neck* of the tooth. The neck of a tooth is *the* part, which, most of all, needs to be kept scrupulously pure and cleanly. This is the point the dentist dreads! A decay at the *neck of a tooth*, anywhere in or under the circumferential margin of the gum, is difficult to arrest under the most favorable circumstances. But when inattention to thorough cleaning at this point becomes habitual, the loss of the affected tooth is well nigh inevitable. Attention to the cervical regions of each tooth is, therefore, an indespensible requisite to perfect dental cleanliness.

The teeth, then, need *careful*, and not "excessive" hygienic regulations. The gums were not made to be "cut through" or torn to pieces by hard brushes and harder rubbing. The super-enamel was not formed to be "worn out" by super-human efforts to keep the tooth clean. The "parencymatous" *gum*, and the "adamantine" *cortex striatus* will bear a great deal of good, vigorous, if not *rigorous* rubbing, to the contrary, notwithstanding. A recorded instance of this fact may be produced. "Geo. Waite, Esq., Surgeon Dentist," relates his own experience as a hard-rubber, in the following passage from his "Critical Inquiry:"

"For the last ten years of my life, I have, perhaps, brushed my teeth and gums harder than any person in England. I may say that I have endeavored to rub them away, but in vain; I have sometimes gone so far as to make them sore, but the result I find is this, that the soreness heals in an almost incredible manner, that the next morning there is a somewhat cartilaginous deposit on them, exuded from the openings of vessels on their surface, sometimes perceptible to the eye, and that when healed there is an increase rather than a decrease."

Such *rigorous* rubbing as this is not essential to dental cleanliness, by any means, yet we are decidedly of the opinion of this dental physiologist, that "nothing is more adapted to giving the teeth and gums strength and a healthy appearance than repeated friction."

We will close this paper with one word about "dentrifices." We never had much faith in dentrifices. We quit their use years ago, and confine ourselves to some suitable "Deutalina" or tooth-wash. We supply our dental friends with a wash which is more suitable to the wants of the teeth and gums than any powders we know of. We have seen that it is on the *necks* of teeth, and little under the margin of the gum, where cleanliness is most needed and the most difficult to be effected. A *wash* will reach these localities better than *powders.* But the objection to dentrifices is this. To have any possible advantage over a wash, a dentrifice must possess a more or less active mechanical property. Any such qualities in a dentrifice are "improper." Whether you rub much or whether you rub moderately, yet a long continued use of a dentrifice is almost sure to "produce," as says Dr. Richardson, "mechanical erosion and inevitable destruction."—*Southern Dental Register.*

WESTMINSTER, Md.

